# Crucial Role for Neuronal Nitric Oxide Synthase in Early Microcirculatory Derangement and Recipient Survival following Murine Pancreas Transplantation

**DOI:** 10.1371/journal.pone.0112570

**Published:** 2014-11-12

**Authors:** Benno Cardini, Katrin Watschinger, Martin Hermann, Peter Obrist, Rupert Oberhuber, Gerald Brandacher, Surawee Chuaiphichai, Keith M. Channon, Johann Pratschke, Manuel Maglione, Ernst R. Werner

**Affiliations:** 1 Center of Operative Medicine, Department of Visceral, Transplant and Thoracic Surgery, Innsbruck Medical University, Innsbruck, Austria; 2 Division of Biological Chemistry, Biocenter, Innsbruck Medical University, Innsbruck, Austria; 3 Department of Anaesthesiology and Critical Care Medicine, Innsbruck Medical University, Innsbruck, Austria; 4 Institute of Pathology, St. Vinzenz Krankenhaus, Zams, Austria; 5 Department of Plastic and Reconstructive Surgery, Johns Hopkins University School of Medicine, Baltimore, Maryland, United States of America; 6 Division of Cardiovascular Medicine, Radcliffe Department of Medicine, Wellcome Trust Centre for Human Genetics, University of Oxford, Oxford, United Kingdom; University of Missouri, United States of America

## Abstract

**Objective:**

Aim of this study was to identify the nitric oxide synthase (NOS) isoform involved in early microcirculatory derangements following solid organ transplantation.

**Background:**

Tetrahydrobiopterin donor treatment has been shown to specifically attenuate these derangements following pancreas transplantation, and tetrahydrobiopterin-mediated protective effects to rely on its NOS-cofactor activity, rather than on its antioxidant capacity. However, the NOS-isoform mainly involved in this process has still to be defined.

**Methods:**

Using a murine pancreas transplantation model, grafts lacking one of the three NOS-isoforms were compared to grafts from wild-type controls. Donors were treated with either tetrahydrobiopterin or remained untreated. All grafts were subjected to 16 h cold ischemia time and transplanted into wild-type recipients. Following 4 h graft reperfusion, microcirculation was analysed by confocal intravital fluorescence microscopy. Recipient survival was monitored for 50 days.

**Results:**

Transplantation of the pancreas from untreated wild-type donor mice resulted in microcirculatory damage of the transplanted graft and no recipient survived more than 72 h. Transplanting grafts from untreated donor mice lacking either endothelial or inducible NOS led to similar outcomes. In contrast, donor treatment with tetrahydrobiopterin prevented microcirculatory breakdown enabling long-term survival. Sole exception was transplantation of grafts from untreated donor mice lacking neuronal NOS. It resulted in intact microvascular structure and long-term recipient survival, either if donor mice were untreated or treated with tetrahydrobiopterin.

**Conclusion:**

We demonstrate for the first time the crucial involvement of neuronal NOS in early microcirculatory derangements following solid organ transplantation. In this model, protective effects of tetrahydrobiopterin are mediated by targeting this isoform.

## Introduction

Ischemia-reperfusion-injury is still a major factor, which negatively influences graft and recipient survival in solid organ transplantation [1], [2]. Especially in pancreas transplantation, ischemia-reperfusion-injury associated pancreatitis with subsequent pro-thrombogenicity is one of the leading causes of early graft failure [3], accounting for the inferior graft survival outcome compared to other abdominal organ transplantations [4]. A hallmark feature in pancreas ischemia-reperfusion-injury is the early microcirculatory breakdown in the transplanted graft, which has been directly associated with the severity of the eventually resulting graft pancreatitis [5].

The two constitutively expressed nitric oxide synthase (NOS) isoforms, the endothelial (eNOS) and the neuronal (nNOS) isoform, play an important role in regulating the vascular tone [6]. Tetrahydrobiopterin (BH4) is an essential co-factor of all NOSs. This compound is structurally related to the vitamins folic acid and riboflavin and is synthesised from guanosine triphosphate in animals and humans [7]. Depletion of BH4 concentrations, e.g. due to oxidative damage, leads to a disturbance of the NOS-BH4 stoichiometry resulting in an “uncoupling” of the enzyme. This term refers to the dissociation of the electron flow from heme iron and to the consequent switch from a NO producing enzyme to an enzyme reducing molecular oxygen to reactive oxygen species resulting, e.g., in vascular dysfunction [8], [9]. This dysfunction can be successfully reversed by BH4 administration [10]–[13] and there is evidence that treatment of hyperlipidaemia [14] and of arterial hypertension [15], two cardiovascular pathologies associated with vascular dysfunction, may act by increasing vascular BH4.

Although eNOS is generally assumed to be the target of BH4 treatment for vascular dysfunction, this assumption has never been unequivocally proven. Beneficial effects were attributed to the endothelial isoform by using the rather unspecific NOS inhibitor *N*-nitro-L-arginine methyl ester (L-NAME) without further dissecting a possible role of the neuronal isoform [16], [17].

In previous studies we have shown, using a mouse model of pancreas transplantation, that administration of BH4 to the donor animal immediately before organ procurement significantly attenuates microvascular injury, thus enabling long-term recipient and graft survival [18]. BH4 is known to date as an essential co-factor of a set of 8 different enzymes including three NOS isoforms, four BH4 dependent aromatic amino hydroxylases (phenylalanine-, tyrosine-, and tryptophan hydroxylase 1 and 2), and the recently described alkylglycerol monooxygenase [19]. However, in this model, the prevention of early microcirculatory derangements with subsequent lethal outcome of the recipient mice has been shown to be NOS dependent. Other BH4-dependent enzymes which have been recently shown to promote tolerance [20] could be excluded as treatment target of BH4 [21].

To elucidate which isoform of NOS is crucially involved in these early microcirculatory derangements and its associated lethal outcome following solid organ transplantation and has therefore to be considered as the target of BH4 therapy, we investigated in the present work donors specifically lacking one of the three NOS isoforms. Herein we demonstrate for the first time the relevance of the neuronal NOS isoform in this deleterious process and identify this enzyme as the major target of BH4 treatment.

## Materials and Methods

### Animals

In a syngeneic pancreas transplantation model, ten- to twelve-week-old male wild type (wt) C57Bl6 (Strain: 000664M), and C57Bl6-based eNOS (Strain: 002684M), nNOS (Strain: 002633M), and iNOS (Strain: 002609M) knockout mice (−/−) obtained from Jackson laboratories (Bar Habor, USA) were used as donor animals. All recipient animals were wt mice. Animals were housed under standard conditions at the animal center of the Innsbruck Medical University, with access to chow and water ad libitum before and after transplantation. Mice received human care in compliance to the “Principles and the Guide for the Care and Use of Laboratory Animals” prepared by the National Academy of Science and published by the National Institutes of Health (NIH Publication No. 86-23, revised 1985). Experiments were approved by the Austrian Federal Ministry for Education, Arts and Culture (BMWF-66.011/0056-II/3b/2011).

### Experimental model

Anesthesia was achieved with an intramuscular injection of 100 mg/kg b.w. ketamine hydrochloride (Ketasol, Dr. E. Graeub, Bern, Switzerland) and 10–15 mg/kg b.w. xylazine (Xylasol, Dr. E. Graeub, Bern, Switzerland). Surgical procedures were performed under clean, but not sterile conditions using an operating microscope with 7–70× magnification (Olympus Inc SZ-STU2, Japan).

Briefly, following midline incision pancreatic grafts were retrieved by detachment of the duodenum, the portal vein and the mesenteric axis. Exocrine secretion was managed by ligation of the choledocho-pancreatic duct. Implantation occurred in the right cervical region. Portal vein and celiac trunk were anastomosed in a non-suture cuff technique to the right external jugular vein and right common carotid artery [22]. Human endpoints (break-off criteria) included weight loss (10%) compared to weight at surgery-date, apathy, hunched back, crippling and intraoperative bleeding. Animals were sacrificed by using terminal isoflurane inhalation.

### Experimental Design

In order to induce severe ischemia-reperfusion-injury, pancreatic grafts were subjected to 16 h cold ischemia time (CIT). Warm ischemia time (WIT) was strictly standardized to 45 min. For graft retrieval and storage the clinically applied perfusion solution Custodiol (HTK, Dr. Franz Köhler Chemie GmbH, Asbach-Hähnlein, Germany) was used [23]. BH4 ((6R)-5,6,7,8-tetrahydro-L-biopterin dihydrochloride) was obtained from Schircks Laboratories, Jona, Switzerland.

The experimental design consisted of 24 groups (n = 5 per group). Confocal intravital fluorescence microscopy and subsequent graft procurement for further analyses were performed at three different time points: before organ recovery (non-transplanted controls), or 2 h, or 4 h following organ reperfusion. Group 1: untreated, non-transplanted wt; group 2: untreated wt 2 h reperfusion; group 3: untreated wt 4 h reperfusion; group 4: BH4 treated, non-transplanted wt; group 5: BH4 treated wt 2 h reperfusion; group 6: BH4 treated wt 4 h reperfusion; group 7: untreated, non-transplanted eNOS−/−; group 8: untreated eNOS−/− 2 h reperfusion; group 9: untreated eNOS−/− 4 h reperfusion; group 10: BH4 treated, non-transplanted eNOS−/−; group 11: BH4 treated eNOS−/− 2 h reperfusion; group 12: BH4 treated eNOS−/− 4 h reperfusion; group 13: untreated, non-transplanted nNOS−/−; group 14: untreated nNOS−/− 2 h reperfusion; group 15: untreated nNOS−/− 4 h reperfusion; group 16: BH4 treated, non-transplanted nNOS−/−; group 17: BH4 treated nNOS−/− 2 h reperfusion; group 18: BH4 treated nNOS−/− 4 h reperfusion; group 19: untreated, non-transplanted iNOS−/−; group 20: untreated iNOS−/− 2 h reperfusion; group 21: untreated iNOS−/− 4 h reperfusion; group 22: BH4 treated, non-transplanted iNOS−/−; group 23: BH4 treated, iNOS−/− 2 h reperfusion; group 24: BH4 treated, 4 h reperfusion.

Donor pretreatment consisted of a single injection of 50 mg/kg b.w. BH4 2 min before pancreas retrieval. Intramuscular administration was chosen due the applicability in small animal models and due to the quick uptake of BH4 observed in previous studies [24].

In addition, 8 groups (n = 5) were used for survival analysis [18]. Group 25: untreated wt graft into wt recipient; group 26: BH4 treated wt graft into wt recipient; group 27: untreated eNOS−/− graft into wt recipient; group 28: BH4 treated eNOS−/− graft into wt recipient; group 29: untreated nNOS−/− graft into wt recipient; group 30: BH4 treated nNOS−/− donor into wt recipient; group 31: untreated iNOS−/− into wt recipient; group 32: BH4 treated iNOS−/− into wt recipient. Observation time consisted of 50 days, providing an independent readout for successful treatment.

### Confocal intravital fluorescence microscopy

Assessment of graft microcirculation by confocal intravital fluorescence microscopy (CIVFM) was performed as described in our previous study [25]. 0.4% fluorescein isothiocyanate (FITC)-labelled dextran (MW 150.000) was purchased from Sigma Aldrich, Vienna, Austria.

### Histopathology

Following fixation in 10% buffered formaldehyde for 24 h, pancreatic tissue samples were embedded in paraffin and slices were stained with hematoxylin and eosin (H&E). The semiquantitative Schmidt pancreatitis score was adopted to quantify parenchymal damage. It describes four categories: edema, acinar necrosis, haemorrhage and fat necrosis, and inflammatory infiltrates [26].

### Immunohistochemistry (IHC)

Paraffin was removed from cut tissue sections by heating them in citrate buffer, pH 6.0. Endogenous peroxidase was blocked with 0.3% hydrogen peroxide and finally washed with Dulbecco's phosphate buffered saline (PBS). Nitrotyrosine-IHC was performed in a diaminobenzidinetetrahydrochloride (DAB) autostainer (DAKO, Copenhagen, Denmark), using an anti-nitrotyrosine rat polyclonal antibody from Upstate Biotechnology (Lake Placid, NY, USA) at 1∶100 dilution. For staining, secondary antibody peroxidase-labeled polymer and 3,3′ DAKO were used. Haemalaun was used for counterstaining. For quantification purposes, the product of proportion of positive cells in quartiles (0, 1, 2, 3, 4), and the staining intensity (0 no staining; 1 weak; 2 moderate; 3 strong) was calculated, yielding a total semiquantitative immunostaining score ranging from 0 to 12.

### Western Blot

To complement IHC findings we additionally performed nitrotyrosine western blots according to a protocol described in our previous publication [18].

### Biopterin tissue levels

Intragraft BH4 concentrations were obtained by a method modified from Fukushima and Nixon [27] as detailed before [18].

Briefly, tissue was homogenized in 5 mM dithioerythrol and incubated with 20 µl 0.5 M HCl and 0.05 M iodine for acidic oxidation or 20 µl 0.5 M NaOH and 0.05 M iodine for basic oxidation for 1 h in the dark. Basic oxidations were then acidified with 20 µl 1 M HCl. All samples were centrifuged and all mixtures received 20 µl 0.1 M ascorbic acid in order to remove excess iodine. Biopterin concentrations were determined by high-performance liquid chromatography (HPLC) using a Nucleosil 10 SA column (250 mm long, 4 mm i.d., Macherey-Nagel, Düren, Germany) and an elution buffer containing 50 mM potassium phosphate buffer, pH 3.0 at a flow rate of 1.5 ml/min. Biopterin was detected by fluorescence detection (excitation 350 nm, emission 440 nm). BH4 concentrations were calculated as difference of biopterin concentration under acidic and basic oxidation conditions. 100 nM biopterin served as standard.

BH2 concentrations were calculated as difference of total biopterin concentration and intragraft BH4 concentration.

### Genotyping

Genotyping was performed according to protocols obtained by the Jackson laboratories using a small portion of mouse-tails. In all nNOS−/− animals enlargement of the stomach due to hypertrophy of the pyloric sphincter was macroscopically visible as described by Huang et al. [28].

### Serum amylase and lipase

Serum amylase and lipase levels were determined in non-transplanted controls and 4 h following reperfusion. Blood samples were analyzed at the Central Institute of Medical and Chemical Laboratory Diagnostics, Innsbruck Medical University. For quantitative pancreatic amylase determination the enzymatic *in-vitro* test P-AMYL (No. 11555812, Cobas, Vienna, Austria) and for lipase determination the enzymatic *in vitro* assay LIP (No 11821733, Cobas, Vienna, Austria) for Roche automated clinical chemistry analysers were used.

### Statistics

Results are expressed as mean ± standard error of the mean (SEM). Statistical analysis was performed using GraphPad Prism 5 (GraphPad Software, La Jolla, CA, USA). Kruskal-Wallis test was used if multiple groups were compared. If statistical significance was achieved, all pairs were compared among each other using the Mann-Whitney-U-test and the Bonferroni post-test. Kaplan-Meier curve was used for survival analyses and groups were compared using the log rank test. A p value of <0.05 was considered to be statistically significant (ns = not significant).

## Results

### Effect of mouse donor genotype and BH4 treatment on early microcirculatory damage

**Figure 1:**
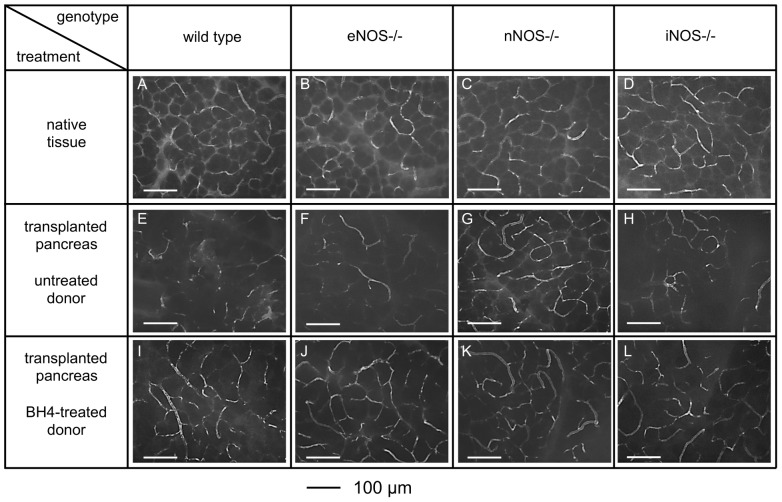
Capillary mesh of pancreata in dependence of donor treatment and donor genotype. Pancreata were taken from BH4-treated or untreated donors with the indicated genotypes, subjected to ischemia, and transplanted to wt recipients of the same background as the knockouts (n = 5 per group). The capillary mesh was stained by infusion with fluorescein labelled dextran and observed by CIVFM. A–D: non-transplanted organs of wt, eNOS−/−, nNOS−/− and iNOS−/−. E–H: organs of untreated donors (wt, eNOS−/−, nNOS−/− and iNOS−/−, respectively), transplanted to wt recipients, 4 h after reperfusion. I–L: organs of donors treated with BH4 (wt, eNOS−/−, nNOS−/− and iNOS−/−, respectively), transplanted to wt recipients, 4 h after reperfusion.

First, we investigated dependence of microcirculatory alterations on donor genotype and BH4 treatment. As depicted in figure 1, representative intravital fluorescence images of non-transplanted pancreatic tissues looked comparable independently from genotype (A–D). Prolonged CIT resulted in a marked breakdown of the microcirculation in untreated, transplanted wt (E), eNOS−/− (F) and iNOS−/− grafts (H). In contrast, following prolonged CIT grafts lacking the neuronal isoform displayed a regular capillary mesh comparable to non-transplanted tissue (G). Donor pre-treatment with BH4 prevented the capillary breakdown independently of the genotype (I–L), except for nNOS−/− where no further improvement in the already well-perfused grafts could be observed (K).

**Figure 2:**
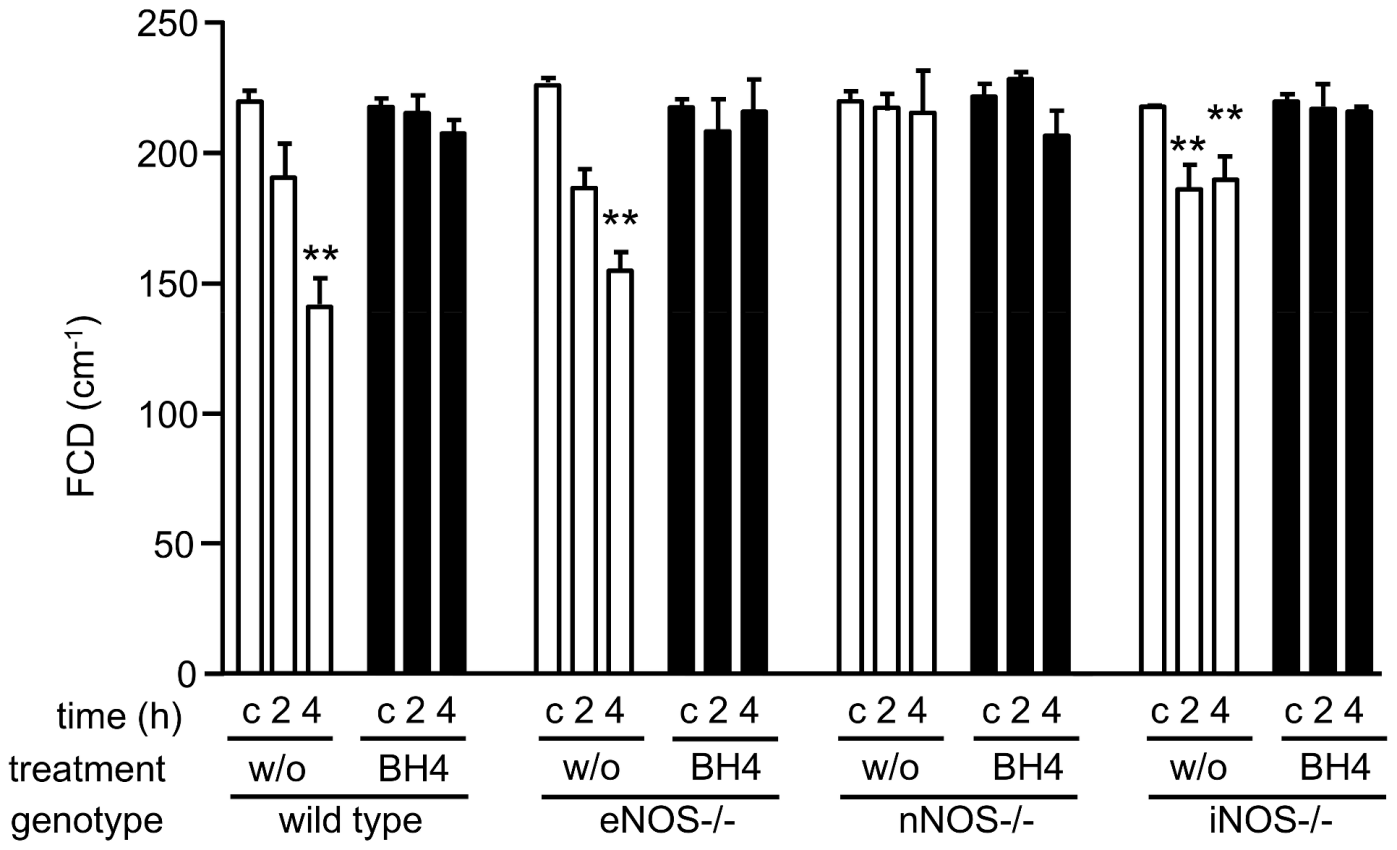
Functional capillary density of pancreata in dependence of treatment, donor type and time point. Pancreata were taken from BH4-treated or untreated donors with the indicated genotypes, subjected to ischemia, and transplanted to wt recipients of the same background as the knockouts (n = 5 per group). FCD was evaluated by digital analysis of CIVFM pictures taken at the indicated time points. c: non-transplanted controls, 2: grafts following 2 h reperfusion, 4: grafts following 4 h reperfusion. Mean values of 5 animals per group +/− SEM are shown. w/o: untreated. Asterisks indicate significant differences between reperfusion time point and the respective non-transplanted control: * p<0.05, ** p<0.01, *** p<0.001.

Mean FCD value (figure 2) in non-transplanted organs (time point c) was 219.07±19.44 in wt and did not differ from knockout strains (p = ns). Exogenous BH4 application did not influence microcirculation of non-transplanted pancreata (p = ns). In contrast, prolonged CIT followed by 2 hours reperfusion resulted in a breakdown of the microcirculation in untreated wt, eNOS−/− as well as iNOS−/− grafts, consistently displaying lower FCD values compared to the respective treated groups, without however reaching statistical significance. FCD values further declined following 4 h reperfusion in untreated wt and eNOS−/− grafts, but not in iNOS−/− grafts, where FCD values did not differ significantly from the previous time point. At this time point, however, FCD levels were significantly lower compared to treated counterparts (untreated wt 4 h vs BH4 treated wt 4 h: p = 0.0007; untreated eNOS−/− 4 h vs BH4 treated eNOS 4 h: p = 0.0027; untreated iNOS 4 h vs BH4 treated iNOS 4 h p = 0.029, respectively). In contrast, nNOS−/− grafts, both, untreated and treated organs, did not show any decrease in FCD values over the entire observation period showing similar FCD values post reperfusion and in non-transplanted controls (p = ns).

### Effect of mouse donor genotype and BH4 treatment on recipient survival

**Figure 3:**
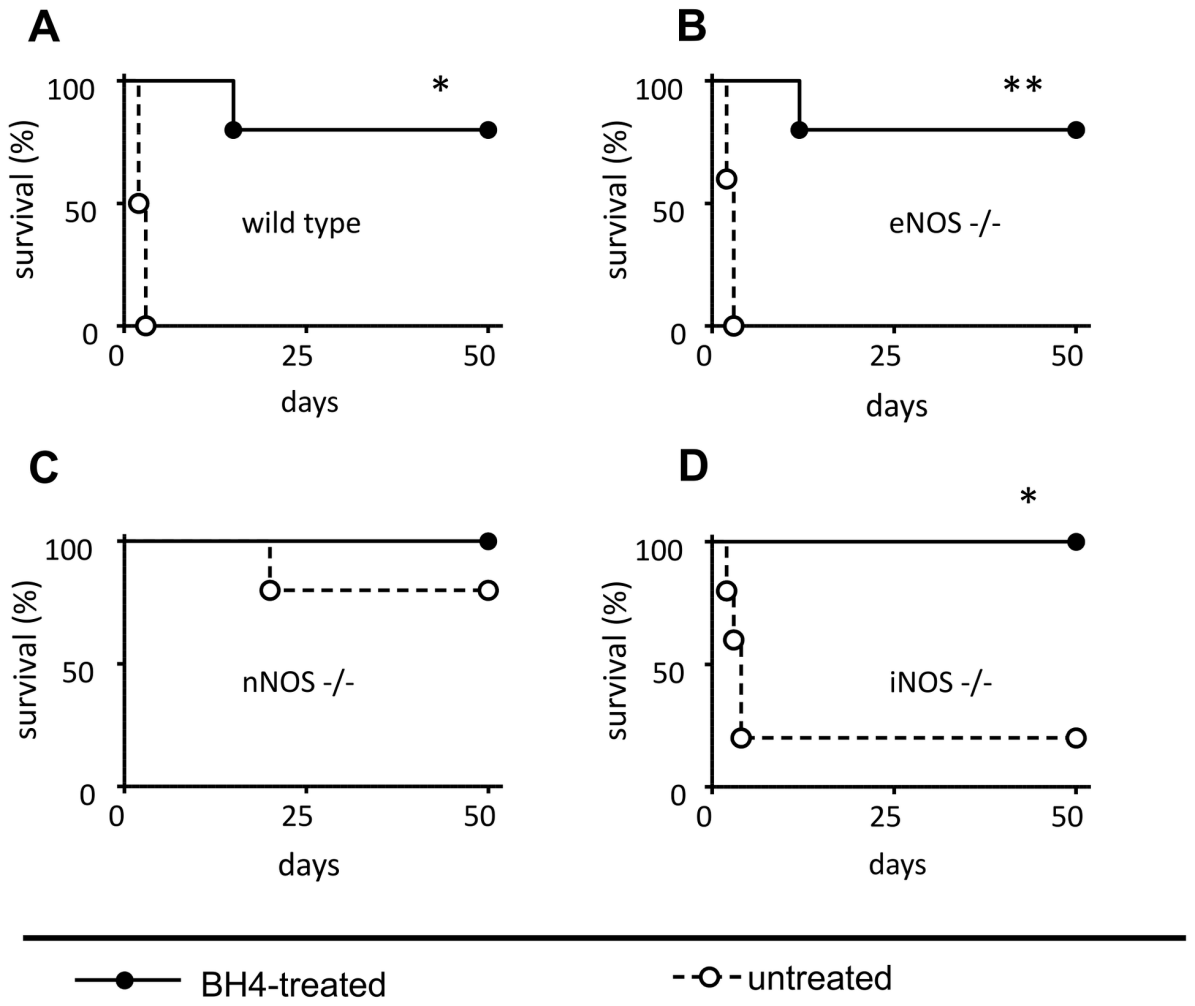
Dependence of recipient survival on donor genotype and treatment status. Pancreata were taken from BH4-treated or untreated donors with the indicated genotypes, subjected to ischemia, and transplanted to wt recipients of the same background as the knockouts. Survival of the animals was monitored for 50 days. Solid line, full circles: Donors treated with BH4. Dashed line, open circles: untreated donor animals. A: wt donors. B: eNOS−/− donors. C: nNOS−/− donors. D: iNOS −/− donors (n = 5 per group). Asterisks indicate significant differences between recipients receiving either grafts from untreated or from BH4 treated donors of the same donor genotype: * p<0.05, ** p<0.01.

We next assessed recipient survival with donor organs from all four mouse strains used in this study with and without BH4 treatment over an observation period of 50 days. Receiving untreated grafts from wt donors (figure 3A) resulted in 100% lethality, with 5 of 5 recipient animals dying within 48–72 h following transplantation. BH4 donor treatment resulted in recipient survival over the whole observation period of 50 days in 4 out of 5 animals and one animal surviving for 15 days (wt untreated vs wt BH4 p = 0.010). With eNOS−/− donors (figure 3B) the results related to recipient survival were essentially the same (eNOS untreated vs eNOS BH4; p = 0.0031). With untreated iNOS−/− donors (figure 3D), 4 of 5 animals died within the first 4 days, whereas one recipient did survive the entire observation period. If iNOS−/− donors were treated with BH4 all 5 recipient animals survived for the entire 50 days observation period (iNOS−/− untreated vs iNOS−/− BH4; p = 0.015). With nNOS−/− donors recipient survival was fundamentally different (figure 3C). In this setting, not only all recipient animals receiving grafts from BH4 treated donors survived the whole observation period but also 4 out 5 recipient animals receiving pancreatic grafts from untreated nNOS−/− donors survived the whole observation period and one died 20 days following transplantation (nNOS−/− untreated vs nNOS BH4; p = ns).

### Effect of mouse donor genotype and BH4 treatment on intragraft BH4 and BH2 levels

**Figure 4:**
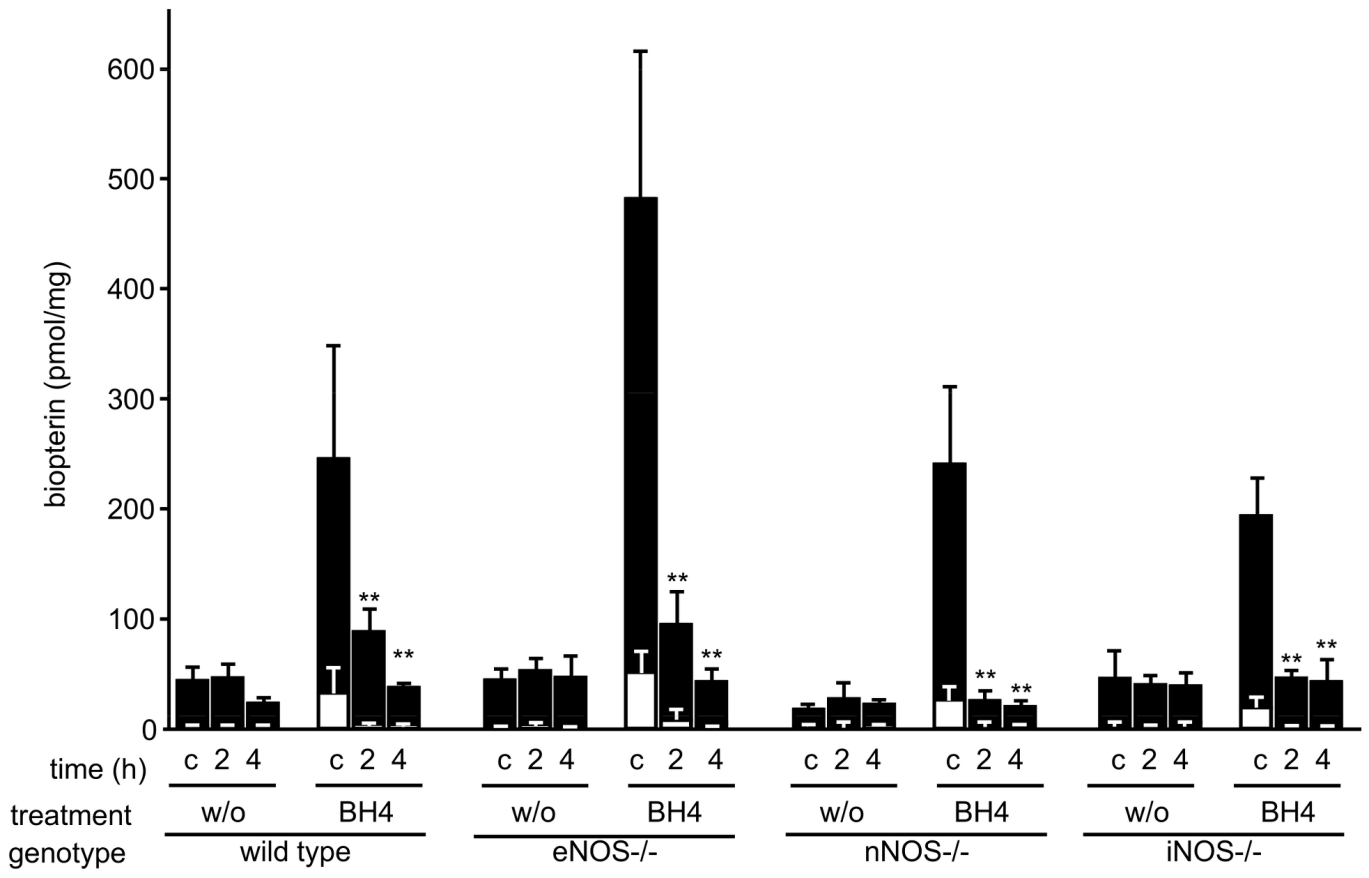
Intragraft biopterin levels in dependence of treatment, donor type and time point. Pancreata were taken from BH4-treated or untreated donors with the indicated genotypes, subjected to ischemia, and transplanted to wt recipients of the same background as the knockouts. Biopterin levels were measured after acidic and alkaline iodine oxidation by HPLC as detailed in the experimental section. Full bars: Tetrahydrobiopterin. Open bars: 7,8-dihydrobiopterin and biopterin. c: non-transplanted controls. 2: grafts following 2 h reperfusion. 4: grafts following 4 h reperfusion. Mean values of 5 animals per group +/− SEM are shown. w/o: untreated. Asterisks indicate significant differences between reperfusion time point and the respective non-transplanted control: ** p<0.01.

Independently from donor genotype, BH4 treatment resulted in an about three- to fivefold increase in intragraft BH4 levels at the time of organ retrieval compared to untreated organs. 2 and 4 h after reperfusion intragraft BH4 levels were back to baseline. Similarly, BH2 levels were significantly higher in treated control animals at the time of organ retrieval and did not differ from untreated animals on later time points (figure 4).

### Effect of mouse donor genotype and BH4 treatment on graft histology

**Figure 5:**
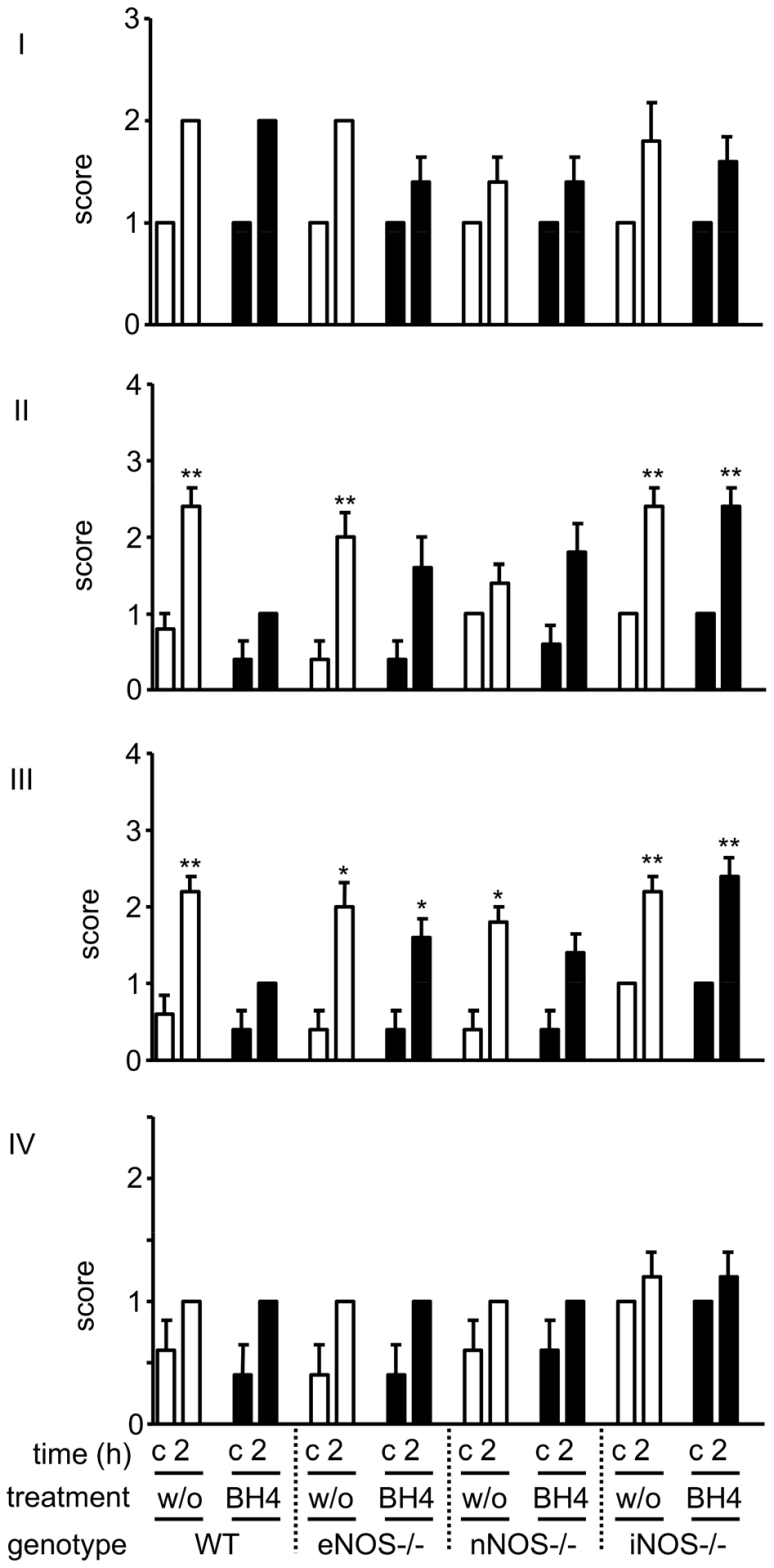
Schmidt pancreatitis score in dependence of donor treatment and donor genotype. The score quantifies parenchymal damage by assessing (I) edema formation, (II) acinar necroses, (III) haemorrhage and fat necroses, and (IV) inflammatory infiltrates. c: non-transplanted controls. 2: grafts following 2 h reperfusion. Mean values of 5 animals per group +/− SEM are shown. w/o: untreated. Asterisks indicate significant differences between reperfusion time point and the respective non-transplanted control: * p<0.05, ** p<0.01.

We then stained paraffin embedded histological sections with H&E and evaluated for edema, acinar necrosis, haemorrhage and fat necroses, and inflammation. Following 2 h reperfusion BH4 treatment significantly reduced acinar necroses as well as haemorrhage and fat necroses in wt compared to the respective untreated wt group (p = 0.0015 and p = 0.0003, respectively). This, however, was not observed in either of the knockout strains (p = ns; figure 5). Following 4 hours reperfusion, results were basically the same with significant differences between untreated and treated animals observed only in wt mice (p = 0.007 respectively), but no differences in knockout-strains (p = ns). Edema and inflammation remained unchanged by BH4 treatment in all strains (p = ns for all). Untreated as well as treated eNOS−/− and treated iNOS−/− displayed significantly lower inflammatory infiltrates compared to wt following 4 h reperfusion (p<0.01, respectively; for the complete dataset see figure S1A and S1B).

### Effect of mouse donor genotype and BH4 treatment on nitrotyrosine formation

Next, we evaluated nitrotyrosine immunohistochemistry as an indirect marker of peroxynitrite production by applying a semiquantitative score. Following 2 h reperfusion, a significant increase in nitrotyrosine formation was observed in untreated wt grafts when compared to the corresponding treated group (p = 0.0003). However, in the knockout groups treatment did not show any significant influence on intragraft nitrotyrosine levels (p = ns). 4 h post reperfusion neither treatment nor genotype affected nitrotyrosine staining in the evaluated grafts (p = ns for all; figure S2A and S2B).

3-nitrotyrosine western blotting did not show any significantly differences in compared groups (figure S3A and S3B).

### Effect of mouse donor genotype and BH4 treatment on amylase and lipase serum levels

**Figure 6:**
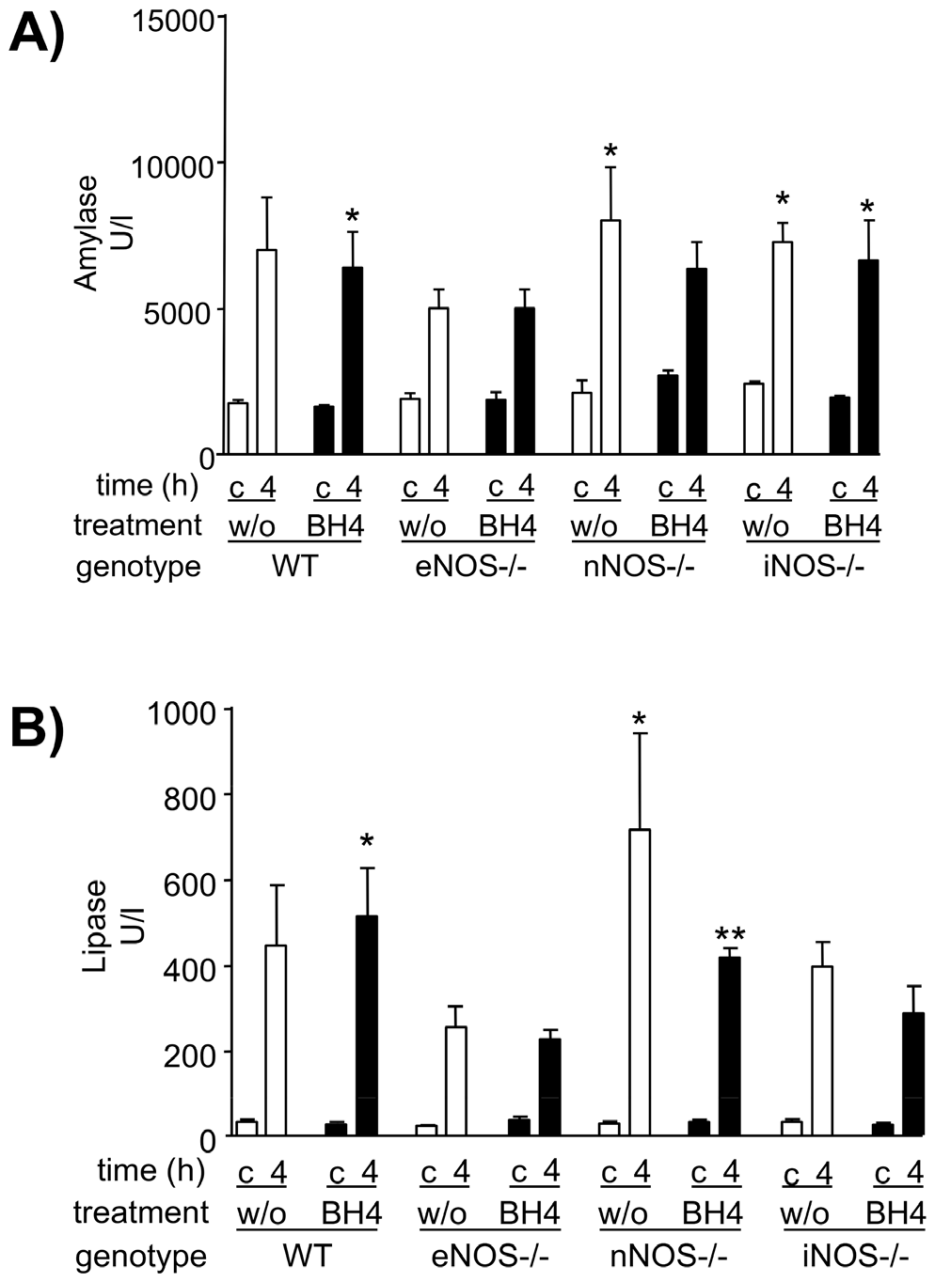
Serum amylase (A) and serum lipase (B) levels in dependence of donor treatment and donor genotype. Serum was taken from BH4-treated or untreated donors with the indicated genotypes, subjected to ischemia, and transplanted to wt recipients of the same background as the knockouts. Enzyme levels were determined by enzymatic *in-vitro* tests for automated clinical chemistry. c: non-transplanted controls. 4: grafts following 4 h reperfusion. Mean values of 5 animals per group +/− SEM are shown. w/o: untreated. Asterisks indicate significant differences between reperfusion time point and the respective non-transplanted control: * p<0.05, ** p<0.01, *** p<0.001.

We also measured amylase and lipase levels in non-transplanted control groups and in grafts following 4 h reperfusion. Baseline amylase and lipase levels in non-transplanted controls did not differ between genotypes and ranged between 1062 U/l and 2987 U/l for serum amylase, and 20 U/l and 70 U/l for serum lipase (p = ns). Figure 6 shows, that both, amylase (A) and lipase (B) levels, were increased if organs were transplanted and reperfused for 4 h. BH4 treatment did not prevent this increase (p = ns for all).

## Discussion

This is the first study showing a critical involvement of the neuronal isoform of NOS in early microcirculatory derangements and subsequent lethal outcome following solid organ transplantation. Using a murine pancreas transplantation model we could demonstrate that the absence of nNOS in the transplanted graft – rather than eNOS or iNOS - not only avoids the breakdown of the capillary perfusion following pancreas reperfusion, but also results in long-term survival in this particular setting where occurrence of early disruption of the capillary mesh in the graft is known to be associated with lethal outcome. In addition, we could clearly demonstrate that it is the missing neuronal isoform in the graft that confers protection to the transplanted organ. Systemic effects mediated through nNOS are not altered in the recipients, since in our study they are all wild types.

These findings highlight important implications like the therapeutic targeting of the organ before transplantation, and the new role of nNOS in early microvascular derangements following solid organ transplantation, clearly overruling the endothelial isoform in this model. Furthermore, the observation that the neuronal isoform mediates the protective effects of BH4 confirms our previous findings that the antioxidant properties of BH4 do not play the major role in preventing the microcirculatory breakdown which anticipates lethal outcome in our pancreas transplantation model [25].

NO is crucially involved in vascular homeostasis. It regulates vascular tone, inhibits platelet and leukocyte aggregation, and smooth muscle cell proliferation. Endogenous NO is primarily generated by the NOS enzyme family, which catalyse in their active dimeric form the reaction of L-arginine and oxygen to L-citrulline and NO [29]. BH4 is essentially required as co-factor for the correct functioning of this reaction donating electrons, stabilising the enzyme, increasing substrate affinity, and scavenging free radicals occurring during NO biosynthesis [7]. Oxidative stress was shown to deplete BH4 resulting in the uncoupling of the endothelial NOS followed by an increased production of reactive oxygen and nitrogen species [9]. Uncoupling of this isoform results in endothelial dysfunction [13] and has been observed in cardiovascular diseases such as atherosclerosis and diabetes mellitus [30], [31]. Based on these results the therapeutic potential of BH4 has been analysed in acute ischemic injuries. Besides the reported protective effects of BH4 treatment in a porcine cardiac ischemia model [32], more recently, in a rat Langendorff model and in a rat kidney clamping model, prevention of ischemia-reperfusion-injury by BH4 treatment has been observed as well [33], [34]. Similarly, in a pig model supplementation of BH4 significantly reduced ischemia-reperfusion-injury following lung transplantation [35].

Even though the NOS isoform primarily investigated in cardiovascular diseases is the endothelial NOS there are several recent *in vitro* as well as *in vivo* studies suggesting that not only the endothelial but also the neuronal isoform is crucially involved in vascular homeostasis by directly influencing the vascular smooth muscle tone [36], [37]. Besides being expressed in neuronal cells of the central nervous system, nNOS has in fact also been detected in cardiac myocytes [38], vascular smooth muscle [39], and endothelial cells [40]. Interestingly, Yamaleyeva et al. have recently shown that inhibition of nNOS reduced salt-induced oxidative stress and renal injury in salt-sensitive rats [41]. This observation fits very well with the hypothesis that nNOS uncoupling rather than eNOS uncoupling due to BH4 depletion is a major mechanism involved in vascular damage. Of note, even though we clearly show that nNOS and not eNOS plays a crucial role in early microcirculatory derangements, our findings do not contradict previous observations, which describe that either BH4 treatment or the NOS inhibitor L-NAME prevent acute pancreatitis. In these studies the action of L-NAME was attributed to inhibition of eNOS [16]. However, L-NAME is an unspecific NOS inhibitor with equal efficacy on nNOS [42]; therefore, a clear differentiation between these two isoforms has never been performed in previous studies.

Intravital microscopy represents a valuable technique to quantify microcirculatory breakdown in experimental as well as in human pancreas transplantation [43], [44]. The significant microcirculatory derangement observed in untreated wt grafts following prolonged CIT is in line with our previous observations and that of other groups [18], [21], [43]. Untreated transplanted eNOS−/− as well as iNOS−/− grafts were similar to wt grafts, with severe disruption of the capillary mesh following reperfusion, unless pretreated with BH4. In contrast, transplanted grafts lacking nNOS displayed a regular capillary pattern and non-affected FCD values even if untreated. Survival analyses closely matched the microcirculatory damage. While transplantation of untreated wt grafts resulted – as expected [18] - in death of the recipient within 3 days, survival over the entire observation period of 50 days could only be achieved if wt donors were treated with BH4. Interestingly, similar results were observed with eNOS−/− and iNOS −/− mice. However, the most striking finding was the survival over the entire observation period of 50 days of animals transplanted with untreated grafts lacking nNOS.

In recipients with iNOS−/− grafts, FCD values decreased from 0 to 2 hours like in wt and eNOS−/− donors, but did not further decrease from 2 to 4 hours. Although survival of iNOS−/− was not significantly different from wt or eNOS−/− it was nevertheless remarkable that one of five untreated animals survived. Comparison to the data obtained with nNOS−/− donors, however, clearly shows that iNOS is only of minor importance as compared to nNOS in our model. We can only speculate about the nature of iNOS contribution. Protection from systemic pathophysiological processes like lethal profound hypotension observed in iNOS−/− animal models of sterile sepsis [45] are unlikely to be involved since it is not the recipient but the donor who carries the knock out. iNOS expression has been observed in endothelium and smooth muscle cells following induction of pancreatitis [46], [47]. The absence of this enzyme in a transplanted graft subjected to a prolonged ischemia time with subsequent graft reperfusion might confer a limited protection.

Measured intragraft BH4 levels show the importance of exogenous BH4 administration to the organ donor. Significant differences between untreated and treated grafts and the direct correlation between high intragraft levels before reperfusion in treated groups and their better survival suggest the treatment target to be in the transplanted organ rather than in the recipient. In accordance with this, the absence of nNOS in the donor and not in the recipient was the important criterion. Even though our model guarantees an exceptional readout by testing recipient survival, the highly active protease and RNAse enzymes present in the pancreas rendered inapplicable protocols established in both our Oxford and Innsbruck laboratories for RNA isolation as well as the study of NOS dimerisation and uncoupling, so that we could not investigate the biochemical basis of the dependence of microcirculatory breakdown on the presence of nNOS in the murine pancreas.

nNOS expression in pancreas has been described in nerve terminals associated with pancreatic acini, excretory ducts as well as blood vessel in numerous mammals including humans and rodents [48]. However, the exact localization of different NOS isoforms as well as defining the specific function still remains a challenge due to difficulties in tissue fixation and preparation [49].

Considering, however, our previous results which clearly exclude that protective effects of BH4 rely on its antioxidative capacity or on other BH4-dependent enzymes rather than on the NOS family [21], in our view, the most plausible explanation is that the observed early microcirculatory derangements are related to the uncoupling of the neuronal NOS in the graft. Hence, we propose the hypothesis that the absence of nNOS in the graft leads to the absence of the deleterious uncoupled nNOS isoform which appears to be a major trigger of the early microcirculatory damage. Presence of the neuronal isoform in wt, eNOS−/− and iNOS−/− grafts requires BH4 administration to avoid this damage and to enable survival.

In contrast to the findings in wt animals, in eNOS, iNOS and nNOS knock-out grafts BH4 pretreatment did not result in a significant decrease of necrotic areas. We observed decreased inflammatory infiltrates in eNOS−/− and iNOS−/− grafts which is in line with Um et al. who demonstrated that the administration of the universal NOS inhibitor L-NAME attenuated the severity of pancreatic damage in an experimental setting [50].

To further test the uncoupling hypothesis we investigated 3-nitrotyrosine formation within pancreatic grafts. 3-nitrotyrosine is an established marker for oxidative damage and peroxynitrite formation. Peroxynitrite has been shown in the presence of uncoupled NOS and it nitrates phenolic rings of tyrosine, leading to the formation of 3-nitrotyrosine [51]. This potent radical is the product of the rapid reaction of NO with oxygen radicals like superoxide and it has been associated with protein oxidation, lipid peroxidation and DNA strand breakage [52]. It is surprising that we could not find any significant differences between treated and untreated groups in both wild type and knockout animals, and this is puzzling since it does not fit with our hypothesis related to the uncoupling of the nNOS. We would have expected significantly less nitrotyrosine formation in grafts of animals showing little microcirculatory derangements but this was not the case. However, since the results so far achieved indicate the prevention of nNOS uncoupling as the most plausible explanation for the observed protective effects of BH4 treatment we suppose shortcomings of the performed analyses – related primarily to the pancreatic tissue full of active enzymes degrading proteins and to a certain point to the semiquantitative character of the analyses – to be a cause of these discrepant observations. We can also not exclude that the selection of an earlier time point would have revealed differences between treated and untreated groups, since the major amount of oxidative burst is expected to occur immediately after reperfusion. In the light of the most important readouts, which are the prevention of microvascular derangements and the long-term survival of the recipients, both, the histopathological as well as immunohistochemical findings at the chosen time points, are probably of minor importance in this study.

Surprisingly, the highly significant results observed in *in vivo* microcirculation analysis and in survival analysis are not supported by serum amylase and serum lipase levels in the recipient. The increase of serum amylase as well as lipase levels is widely accepted to indicate acinar cell necroses and subsequent tissue damage in pancreatic organs. This is regularly observed during acute pancreatitis as well as following pancreas transplantation. In our study, both parameters were elevated following 4 h reperfusion, showing however no differences between untreated and treated groups, independently of their genotype. This indicates that the knock out of the neuronal NOS isoform could not prevent a certain degree of early reperfusion-associated pancreatitis. We assume that the observed discrepancy between high pancreas enzyme levels in the serum and intact microcirculation in nNOS −/− graft recipients is related to the time point chosen for this analysis, probably too early to detect any differences. This fits very well with a clinical observation in pancreas recipients where microcirculatory derangements observed by *in vivo* orthogonal polarized spectral (OPS) imaging during the first 30 minutes of graft reperfusion did not correlate with amylase/lipase levels in the serum during the first 48 h following reperfusion. A significant inverse correlation (low initial capillary reperfusion and corresponding high pancreatic enzyme levels in the serum) could only be detected as late as from day 3 on post transplantation [44]. We suppose therefore that later time points would probably have shown a settling of the pancreatitis parameters in animals surviving the entire observation period. However, comparison between different groups in this model is possible only up to 48 h–72 h following transplantation due to non-survivors, and this time point would still have probably been too early to show any differences.

In conclusion, this study clearly shows for the first time that the neuronal isoform of NOS is critically involved in early microcirculatory derangements and its associated lethal outcome following solid organ transplantation. Robust readouts like *in vivo* microcirculatory assessment and survival analysis of the recipient animals support the neuronal NOS as a novel therapeutic target in ischemia-reperfusion-injury.

## Supporting Information

Figure S1 (**A**) **Graft histopathology in dependence of donor treatment and donor genotype.** Pancreata were taken from BH4-treated or untreated donors with the indicated genotypes, subjected to ischemia, and transplanted to wt recipients of the same background as the knockouts. Pancreas specimens were embedded in paraffin and slices were stained with H&E. I–IV: non-transplanted organs of wt, eNOS−/−, nNOS−/− and iNOS−/−. V–VIII: organs of untreated donors (wt, eNOS−/−, nNOS−/− and iNOS−/−, respectively), transplanted to wt recipients, 2 h after reperfusion. IX–XII: organs of donors treated with BH4 (wt, eNOS−/−, nNOS−/− and iNOS−/−, respectively), transplanted to wt recipients, 2 h after reperfusion. (**B**) **Schmidt pancreatitis score in dependence of donor treatment and donor genotype.** The score quantifies parenchymal damage by assessing (I) edema formation, (II) acinar necroses, (III) haemorrhage and fat necroses, and (IV) inflammatory infiltrates. c: non-transplanted controls. 2: grafts following 2 h reperfusion. 4: grafts following 4 h reperfusion. Mean values of 5 animals per group +/− SEM are shown. w/o: untreated.
(TIF)

Figure S2 (**A**) **Graft nitrotyrosine IHC in dependence of donor treatment and donor genotype.** Pancreata were taken from BH4-treated or untreated donors with the indicated genotypes, subjected to ischemia, and transplanted to wt recipients of the same background as the knockouts. Anti-nitrotyrosine rat polyclonal antibody, with a secondary peroxidase-labelled antibody for detection, was used. Haemalaun blue or methyl green was used for counterstaining. I–IV: non-transplanted organs of wt, eNOS−/−, nNOS−/− and iNOS−/−. V–VIII: organs of untreated donors (wt, eNOS−/−, nNOS−/− and iNOS−/−, respectively), transplanted to wt recipients, 2 h after reperfusion. IX–XII: organs of donors treated with BH4 (wt, eNOS−/−, nNOS−/− and iNOS−/−, respectively), transplanted to wt recipients, 2 h after reperfusion. (**B**) **Semiquantitative IHC score in dependence of donor treatment and donor genotype.** The product of the proportion of positive cells in quartiles and the staining intensity was calculated yielding a total score ranging from 0 to 12. c: non-transplanted controls. 2: grafts following 2 h reperfusion. 4: grafts following 4 h reperfusion. Mean values of 5 animals per group +/− SEM are shown. w/o: untreated.
(TIF)

Figure S3 (**A**) **Representative 3-nitrotyrosine and α-actin western blot in dependence of donor treatment and donor genotype.** Pancreata were taken from BH4-treated or untreated donors with the indicated genotypes, subjected to ischemia, and transplanted to wt recipients of the same background as the knockouts. Mouse monoclonal antibody to 3-nitrotyrosine was used. Staining with mouse monoclonal anti-actin antibody was performed as control for protein loading. Detection was done using ECL Plus detection reagent and membranes were scanned with Typhoon scanner. c: non-transplanted controls. 2: grafts following 2 h reperfusion. 4: grafts following 4 h reperfusion. w/o: untreated. (**B**) **3-nitrotyrosine to α-actin ratio in dependence of donor treatment and donor genotype.** Image Quant TL software was used for quantification of the band, evaluation occurred by comparison of the determined 3-nitrotyrosine to α-actin ratio. c: non-transplanted controls. 2: grafts following 2 h reperfusion. 4: grafts following 4 h reperfusion. Mean values of 5 animals per group +/− SEM are shown. w/o: untreated. For significances of difference, see text. w/o: untreated.
(TIF)
