# Supplementary figures and images for: Crucial Role for Neuronal Nitric Oxide Synthase in Early Microcirculatory Derangement and Recipient Survival following Murine Pancreas Transplantation
Source: PLoS One. 2014 Nov 12;9(11):e112570. doi: 10.1371/journal.pone.0112570 (PMC4229216; doi:10.1371/journal.pone.0112570)

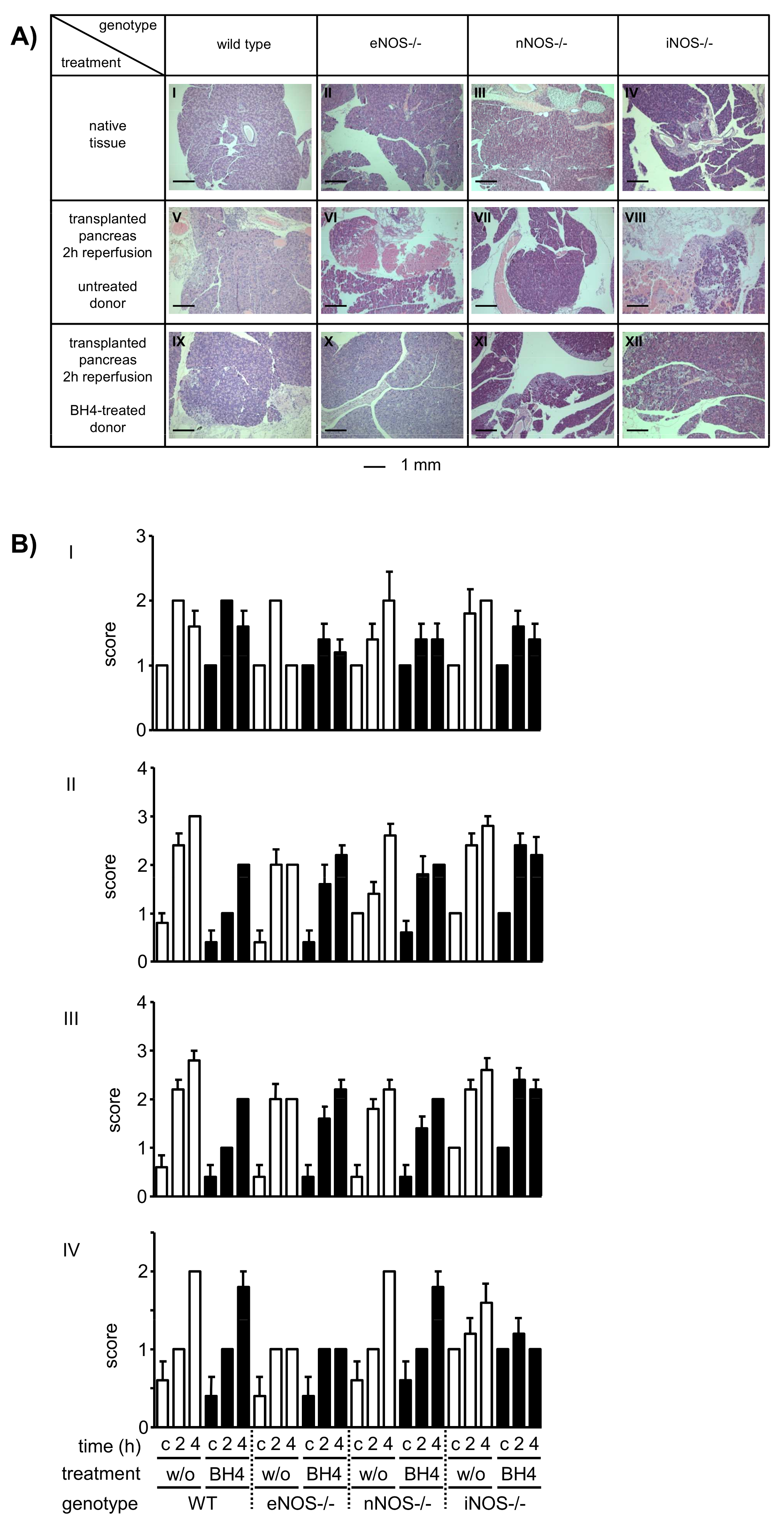

Supplement: Figure S1 — (A) Graft histopathology in dependence of donor treatment and donor genotype. Pancreata were taken from BH4-treated or untreated donors with the indicated genotypes, subjected to ischemia, and transplanted to wt recipients of the same background as the knockouts. Pancreas specimens were embedded in paraffin and slices were stained with H&E. I–IV: non-transplanted organs of wt, eNOS−/−, nNOS−/− and iNOS−/−. V–VIII: organs of untreated donors (wt, eNOS−/−, nNOS−/− and iNOS−/−, respectively), transplanted to wt recipients, 2 h after reperfusion. IX–XII: organs of donors treated with BH4 (wt, eNOS−/−, nNOS−/− and iNOS−/−, respectively), transplanted to wt recipients, 2 h after reperfusion. (B) Schmidt pancreatitis score in dependence of donor treatment and donor genotype. The score quantifies parenchymal damage by assessing (I) edema formation, (II) acinar necroses, (III) haemorrhage and fat necroses, and (IV) inflammatory infiltrates. c: non-transplanted controls. 2: grafts following 2 h reperfusion. 4: grafts following 4 h reperfusion. Mean values of 5 animals per group +/− SEM are shown. w/o: untreated. (TIF) [file pone.0112570.s001.tif]

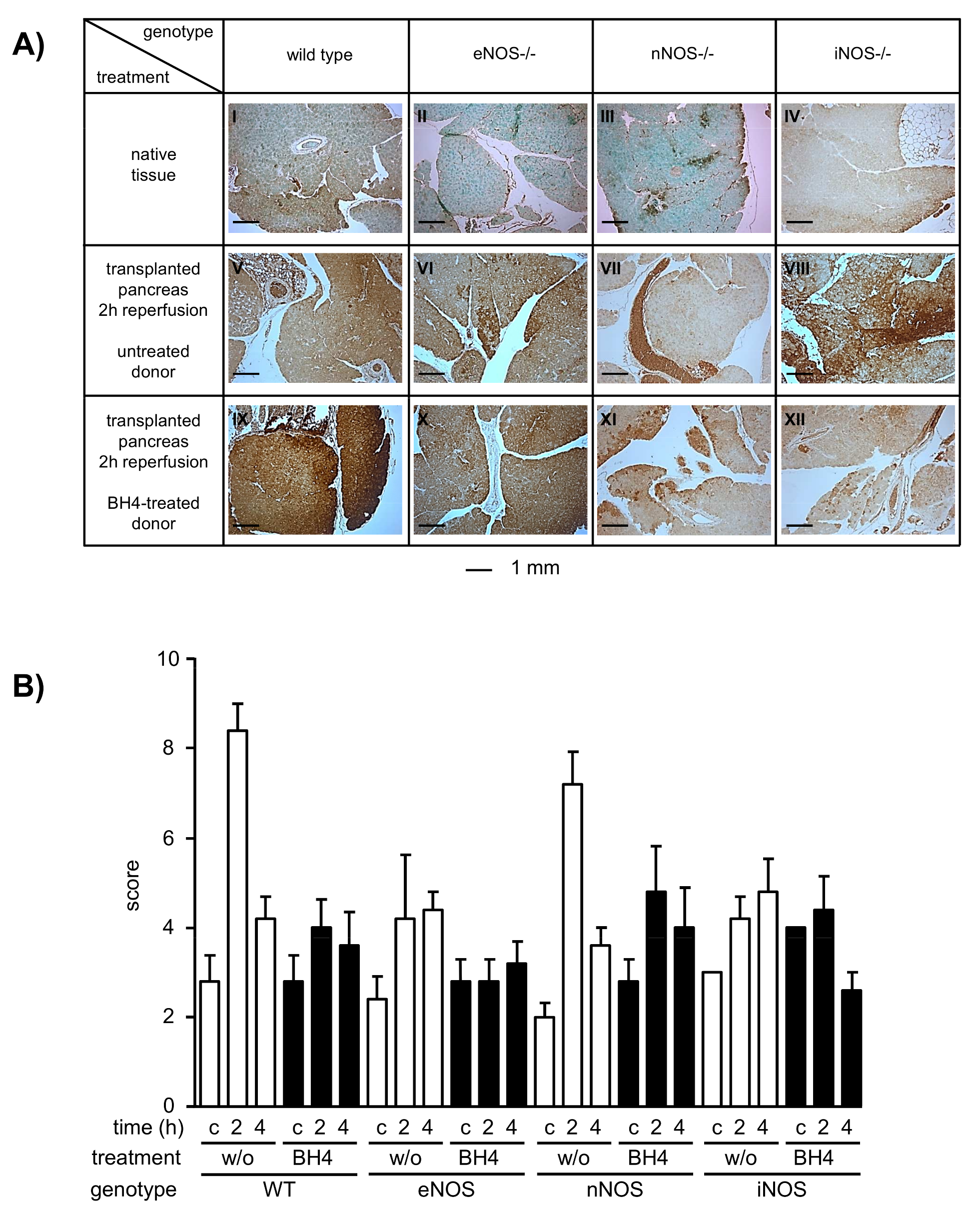

Supplement: Figure S2 — (A) Graft nitrotyrosine IHC in dependence of donor treatment and donor genotype. Pancreata were taken from BH4-treated or untreated donors with the indicated genotypes, subjected to ischemia, and transplanted to wt recipients of the same background as the knockouts. Anti-nitrotyrosine rat polyclonal antibody, with a secondary peroxidase-labelled antibody for detection, was used. Haemalaun blue or methyl green was used for counterstaining. I–IV: non-transplanted organs of wt, eNOS−/−, nNOS−/− and iNOS−/−. V–VIII: organs of untreated donors (wt, eNOS−/−, nNOS−/− and iNOS−/−, respectively), transplanted to wt recipients, 2 h after reperfusion. IX–XII: organs of donors treated with BH4 (wt, eNOS−/−, nNOS−/− and iNOS−/−, respectively), transplanted to wt recipients, 2 h after reperfusion. (B) Semiquantitative IHC score in dependence of donor treatment and donor genotype. The product of the proportion of positive cells in quartiles and the staining intensity was calculated yielding a total score ranging from 0 to 12. c: non-transplanted controls. 2: grafts following 2 h reperfusion. 4: grafts following 4 h reperfusion. Mean values of 5 animals per group +/− SEM are shown. w/o: untreated. (TIF) [file pone.0112570.s002.tif]

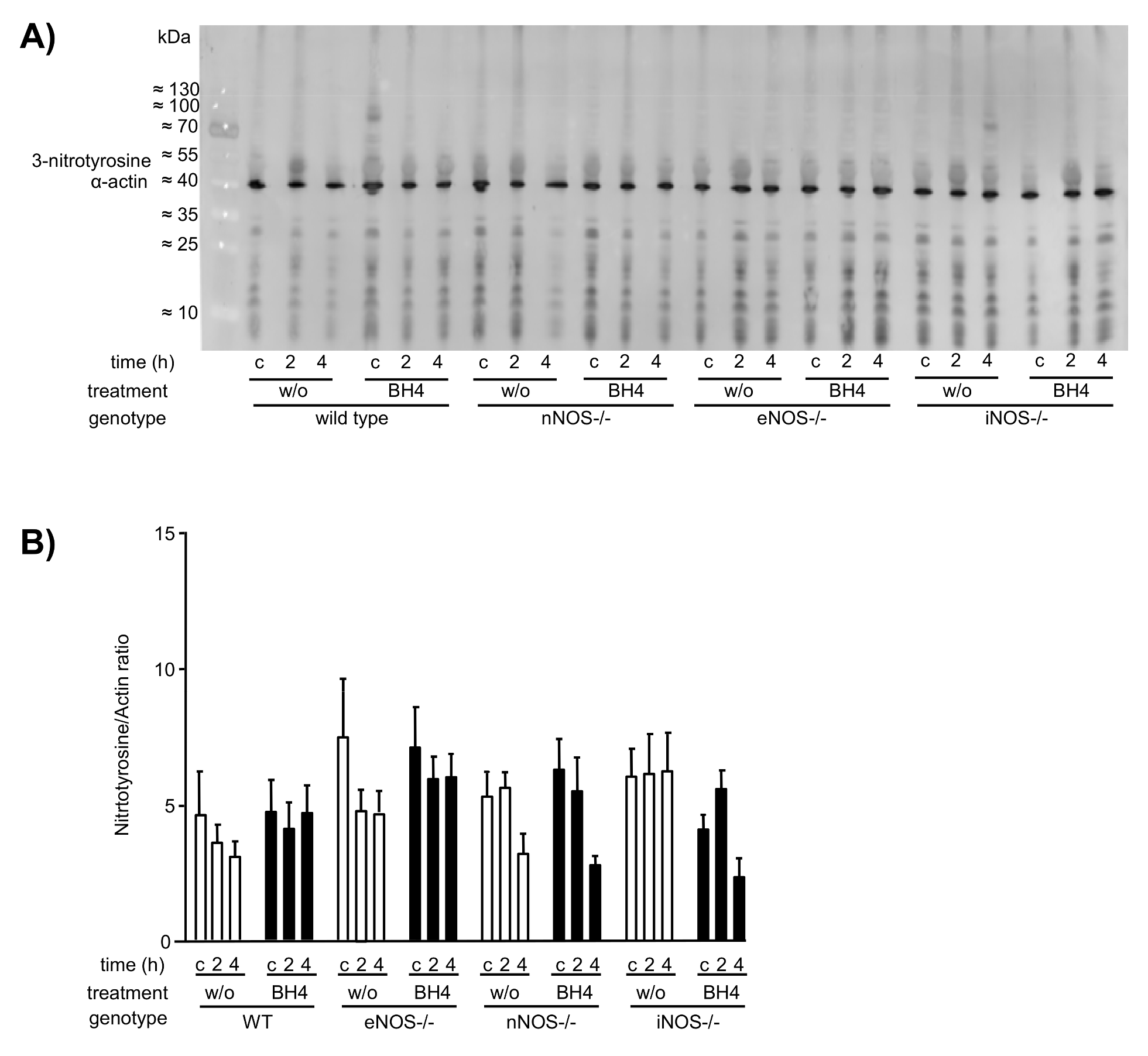

Supplement: Figure S3 — (A) Representative 3-nitrotyrosine and α-actin western blot in dependence of donor treatment and donor genotype. Pancreata were taken from BH4-treated or untreated donors with the indicated genotypes, subjected to ischemia, and transplanted to wt recipients of the same background as the knockouts. Mouse monoclonal antibody to 3-nitrotyrosine was used. Staining with mouse monoclonal anti-actin antibody was performed as control for protein loading. Detection was done using ECL Plus detection reagent and membranes were scanned with Typhoon scanner. c: non-transplanted controls. 2: grafts following 2 h reperfusion. 4: grafts following 4 h reperfusion. w/o: untreated. (B) 3-nitrotyrosine to α-actin ratio in dependence of donor treatment and donor genotype. Image Quant TL software was used for quantification of the band, evaluation occurred by comparison of the determined 3-nitrotyrosine to α-actin ratio. c: non-transplanted controls. 2: grafts following 2 h reperfusion. 4: grafts following 4 h reperfusion. Mean values of 5 animals per group +/− SEM are shown. w/o: untreated. For significances of difference, see text. w/o: untreated. (TIF) [file pone.0112570.s003.tif]
